# The effectiveness of e-learning in focused cardiac ultrasound training: a prospective controlled study

**DOI:** 10.1186/s12909-025-07409-y

**Published:** 2025-05-30

**Authors:** Johannes Ruppert, Rebecca Krüger, Sebastian Göbel, Susanna Wolfhard, Liv-Annebritt Lorenz, Andreas Michael Weimer, Roman Kloeckner, Elias Waezsada, Holger Buggenhagen, Julia Weinmann-Menke, Johannes Matthias Weimer

**Affiliations:** 1 Department of Medicine, Justus Liebig University, Giessen, Germany; 2 Rudolf Frey Learning Clinic, University Medical Center of the Johannes Gutenberg University Mainz, Mainz, Germany; 3 Rehabilitation Center Bayerisch Gmain, Bayerisch Gmain, Germany; 4 Department of Medicine, Eberhard Karls University, Tübingen, Germany; 5 Department of Radiation Oncology and Radiotherapy, University Medical Centre of the Johannes Gutenberg University Mainz, Mainz, Germany; 6 Center of Orthopedics, Trauma Surgery, and Spinal Cord Injury, Heidelberg University Hospital Heidelberg, Heidelberg, Germany; 7 Institute of Interventional Radiology, University Hospital Schleswig-Holstein - Campus Lübeck, Lübeck, Germany; 8 Clinic for Electrophysiology, Heart and Diabetes Center NRW, Faculty of Medicine, Ruhr-University Bochum, OWL (University of Bielefeld), Bad Oeynhausen, Germany; 9 Department of Internal Medicine I, University Medical Center of the Johannes Gutenberg University Mainz, Mainz, Germany

**Keywords:** E-Learning, Focused cardiac ultrasound, Competency-Based education, Blended learning, Ultrasonography, Medical education

## Abstract

**Introduction:**

Focused Cardiac Ultrasound (FOCUS) is an essential tool for rapid cardiac assessment across various clinical subspecialties. Consequently, teaching foundational FOCUS skills is of critical importance. This study investigates the effectiveness of e-learning in imparting FOCUS skills.

**Materials and methods:**

This prospective, controlled study assessed competency development among medical students attending a FOCUS workshop (study group) at two time points: T1 (pre-training) and T2 (post-training, after completing e-learning). The competence gain of the group was compared to a reference group (control group) of physicians who had also used the e-learning in preparation for a certified FOCUS workshop. Objective competencies were measured at both time points using short-answer and multiple-choice theory tests. Subjective self-assessments of competencies and opinions of the e-learning were investigated through evaluation forms at T2 using a 7-point Likert scale (1 = strongly disagree, 7 = strongly agree). Demographic information was collected at T1, and user behaviour during e-learning was assessed at T2. Primary endpoints were the increase in theoretical competencies (study group) and the comparison of subjective and objective competency levels (study vs. reference).

**Results:**

A total of 104 participants (study group = 48; reference group = 56) were included. The study group exhibited a significant (*p* < 0.001) increase in theoretical competencies. However, at T2 the reference group achieved significantly higher theoretical test scores (*p* < 0.001). One influencing factor was previous practical experience (*p* = 0.02), which was significantly higher in the reference group (*p* < 0.001). Both groups estimated their competency at the end of preparation to be at similar levels (4.3 ± 0.9 scalepoints [SP] versus 4.3 ± 1.0 SP; *p* = 0.94). Evaluation results of the e-learning were positive in both groups (5.8 ± 0.9 SP versus 6.2 ± 0.7 SP; *p* = 0.04), with results in the reference group being significantly higher.

**Conclusion:**

Both the improvement in competencies and the positive reception of digital learning media should encourage the increased implementation of e-learning formats. This study shows that such formats in ultrasound training can effectively complement face-to-face workshops and should be included in certified training curricula.

**Supplementary Information:**

The online version contains supplementary material available at 10.1186/s12909-025-07409-y.

## Introduction

### Digitalisation in sonography training

Digital media and training have profoundly changed medical education [1]. Clinical training now often involves the use of web-based learning platforms or software [1, 2], and digital media is used increasingly widely in clinical settings or for educational purposes [3]. The COVID-19 pandemic further reinforced this development [4–7]. With in-person lectures almost impossible, online formats became vital to ongoing teaching and learning provision, thereby increasing awareness and use of innovative digital teaching methods and media [5, 7–9].

Accordingly, ultrasound teaching increasingly uses a combination of digital and in-person education models (so-called “blended learning”), which are currently being developed and further assessed in research [10–14]. Along with traditional educational media (e.g. books, scripts, slides) and teaching methods (e.g. in-person lectures, small group in-person courses), new “blended learning” models also incorporate digital educational media (such as e-learning) and teaching methods (such as video-based education, tele-education, and webinars) in instruction [1, 12–19]. Present studies suggest that digital educational media and teaching methods are positively received by learners and can effectively aid and complement training and competency development [1, 3, 8, 13, 14, 17, 20].

E-learning, defined as the use of digital media for educational purposes [5], has broadened the range of teaching methods available not only in general medical training, but in the teaching of ultrasound in particular [1–3, 8, 10, 14, 17, 20]. E-learning can take many varying forms, from videos of lectures to small-group teaching videos, and from digital reference works, pictorial or video atlases, e-books, and animated 3D models, to fully interactive online courses that combine several or all of these media [1, 3, 5, 7, 8, 14, 17]. Therefore, it is unsurprising that existing e-learning concepts in medical educational research vary immensely regarding structure, duration, and quality of educational offerings [2, 3, 10, 17]. As a result, recommendations such as the World Interactive Network Focused on Critical Ultrasound (WINFOCUS) or other recommendations for the implementation of e-learning in sonography training emphasise structured competency-based training and are often the basis for many sonography training programmes worldwide [21–23]. In this context, e-learning can support theoretical and practical competency development [2, 13, 24–34]. This development has already been evidenced in the teaching of abdominal ultrasound [14, 18, 19], MSK sonography [35], head and neck ultrasound [31–33], and emergency ultrasound [2, 34, 36].

Blended learning and e-learning courses have also been used to teach Focused Cardiac Ultrasound (FOCUS) [2, 25, 27–30, 37, 38]. These digital methods have been used to impart theoretical competencies like cardiac anatomy and physiology, to promote the acquisition of practical competencies, and to deepen and revise newly learned course content [25, 30, 39].

Physicians often use FOCUS in clinical settings, especially when making case-specific clinical decisions or in emergencies [40, 41]. Early training in FOCUS is therefore important, as it provides foundational knowledge of cardiac anatomy and sonography, reinforces anatomical and sonographic skills through interactive content, and preparing students effectively for their future clinical work [28, 30].

For ultrasonography in general and FOCUS in particular, an understanding of anatomical spatial relationships, the precise handling or movement of the probe, and visual-spatial ability are important skills [42–45]. The development of these and other competencies is often supported by in-person instruction and guidance by professionals during courses. Nevertheless, pre-course preparation can improve course competency gain by making the most effective and efficient use of the in-person course time [20, 30]. Specifically, beginners can benefit from high-quality e-learning to learn the basics of complex cardiovascular imaging before a FOCUS course [20, 30, 41].

### Research problem and aim

While specialist societies and panels of experts have promoted e-learning and increasingly integrated it into the teaching of sonography, there are no shared quality standards, accreditation methods, or formal certification processes for e-learning formats [10, 20, 41]. More evidence regarding the effective use of e-learning should suggest ways of achieving this formalisation of e-learning instruction [10], particularly for the teaching of FOCUS [39]. Although previous studies have explored the effectiveness and acceptance of e-learning among different user groups, such as students and physicians, usually this has been done in isolation, comparing only one user group with a non-intervention control group [2, 9, 24, 25, 28, 30, 37, 41, 46, 47]. To our knowledge, no existing study compares the competencies and perceptions of both students and physicians learning FOCUS through the same e-learning platform. Due to their different levels of previous exposure to echocardiography, it was decided to include medical students and physicians as a participant in the study. For medical students, FOCUS training provides an opportunity to acquire theoretical knowledge and basic skills in echocardiography, filling a gap in traditional curricula and preparing them for later, more advanced training. Physicians, with their clinical experience, provide a meaningful reference group to assess the effectiveness of e-learning in both novice and experienced learners and to identify areas for optimization. Unlike previous studies that have focused solely on either medical students or practicing physicians [2, 9, 24, 25, 28, 30, 37, 41, 46, 47], our study compares both groups to explore how prior clinical experience affects the effectiveness of e-learning in FOCUS training. This comparison will provide insight into whether e-learning is equally effective for learners with different levels of clinical exposure. Our study addresses this gap by comparing competency development through e-learning between students and physicians, using the latter as a reference for skill acquisition.

The primary aim of this study was to investigate the effectiveness of e-learning as a preparatory tool for a FOCUS course, specifically in enhancing theoretical knowledge acquisition among medical students. To contextualize competency gain across different levels of clinical experience, a group of physicians served as a reference (control) group. By comparing both groups, the study explores how well e-learning supports learners with varying baseline competencies in preparing for a structured ultrasound training. Secondary aims of the study are to evaluate the quality of FOCUS-specific e-learning and the general acceptance of e-learning in sonography education. The results offer valuable evidence to support the development of digital teaching and optimise future teaching concepts.

## Materials and methodology

### Study design, participant (recruitment) and study procedure

**Fig. 1:**
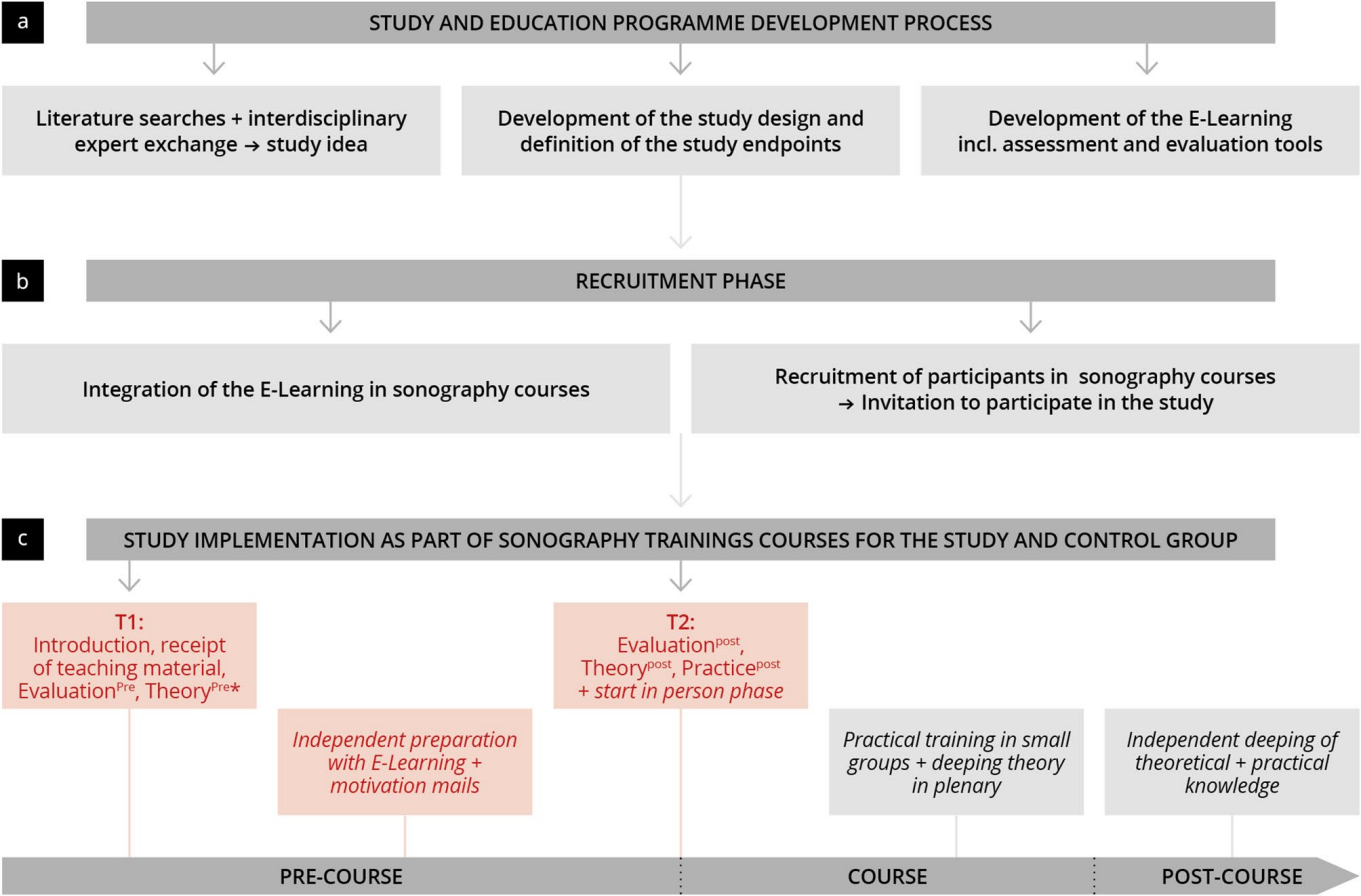
Chronological representation of the whole study development and procedure including time for data collection. After the study and the training program were designed **(a)**, participants were recruited **(b)** to take part in the study through the workshop **(c)**, * Reference group did not complete the T1 theory test

This prospective, controlled observational trial (Fig. 1) was conducted from 2022 to 2023 [48]. The aim was to investigate the effectiveness of an e-learning-assisted preparation period before an in-person FOCUS workshop. We recruited medical students during their clinical years (study group) to participate in a voluntary extracurricular workshop called “Focused Cardiac Ultrasound (FOCUS)” [45]. A second group of physicians participating in a certified workshop were recruited as controls (reference group) [49]. Blended learning served as the didactic approach for this workshop [50]. Participants were recruited via official email invitations: the study group through a mailing list from the university’s Office for Student Affairs, and the reference group via the portal of a specialist society. Both groups received full access to the e-learning module, which they were expected to complete before the FOCUS workshop. Data was collected over the duration of 4 workshops (2 for students and 2 for physicians). At two different times (T1 (*pre)* = before the commencement of the workshop; T2 (*post)* = after the preparation period but before the in-person workshop), we collected written and digital evaluation forms from the groups (evaluation_pre_, evaluation_post_) to register demographic backgrounds and user behaviour. Additionally we carried out two theory tests (study group: theory_pre_, theory_post_ and reference group: theory_post_) to analyse the effectiveness of the e-learning [51, 52]. The physicians in the reference group did not complete the theory_pre_, as they were experienced professionals already familiar with ultrasound examinations, to minimise potential bias. The theory_post_ was used for evaluation purposes only and was not a prerequisite for participation in the workshop. Data collection was completed prior to the practical phase of the workshop. Inclusion criteria for participants were successful completion of the first state examination, usage of educational materials, and full participation in evaluations and tests. Primary endpoints were defined as an objective increase in theoretical competencies (study group), and subjective and objective level of competency (study versus reference group) surveyed in the evaluations and theory-tests, respectively. Secondary endpoints were defined as the evaluation of the e-learning and assessed acceptance, assessed by evaluations.

### Process of the preparation period for the workshop

As typical in blended-learning teaching approaches, the workshop was split into a pre-course phase (a digital introduction and self-guided preparation with the educational medium), a course phase (in-person theoretical and practical training), and a post-course phase (a self-guided wrap-up) [50, 53]. The data collection times can be found in the study design and Fig. 1.

After registration for the workshop and one introductory plenary session (T1) participants of the study group received access to the e-learning and instructions on how to complete the digital tasks. Participants were given one to two weeks for digital preparation.

In parallel, the reference group also received access to the same e-learning content and a detailed preparation guide upon registration and digital completion of T1. Their preparation period varied from two weeks to up to four months, depending on the registration date. To sustain the motivation for the e-learning, reminder emails were sent throughout the preparation phase.

At the beginning of the in-person workshops both groups completed T2 [51].

Subsequently, theoretical and practical teaching sessions were conducted in small groups of 4–5 participants, each supervised by an experienced instructor. Practical sessions involved hands-on training with standardized patient models, focusing on probe positioning, image acquisition, and interpretation. Participants had continued access to the e-learning module throughout the study period, including after the initial pre-workshop phase, allowing for further self-guided revision.

The workshop consisted of 16 teaching units of 45 min based on pre-existing concepts [21, 45, 49]. Fundamental were the section planes proposed by the WINFOCUS for transthoracic ultrasound [22]. Data was collected until the end of the preparation period, i.e., the beginning of the workshop.

### Structure of the FOCUS E-learning

To align with the defined learning goals of the course concept [21, 45, 49] and the recommendations of specialist societies [21, 22, 54], the e-learning was designed by ultrasound experts, didactics, and qualified IT specialists. The design of the e-learning was driven by the need to ensure content of high quality, specifically aligned with the course objectives and informed by established best practices in the field. The development and implementation of the e-learning followed the waterfall model, systematically refining the project through sequential phases to ensure thorough updates at each stage [55]. The structure of the e-learning course can be found in Supplement 1 and exemplary extracts are provided in Supplements 2–3. The web-based e-learning was accessed via an internet browser. Like the subsequent course, the e-learning was conducted in German. It was structured into two parts: ‘basics’, covering ultrasound physics, image production, and topographic anatomy; and ‘cardio-specific learning’, covering multiple, similarly structured chapters on standard section planes, tips for probe handling, plane orientation and optimisation, sonoanatomy, and examination questions and protocols. Pathologies were not taught. Individual revision cards (‘slides’) built the foundation of the e-learning. Educational content could be viewed in the form of continuous texts, bullet points, graphics, and/or video clips, all of which were designed to make the educational content more accessible and to clarify the examination protocol. Tables could be filled in and graphics labelled as further interactive features.

### Test and evaluation instruments

The conceptual design of the test format and evaluation form resulted from the consensus of ultrasound and didactics experts according to currently available research and to meet the learning goals of the workshop [21, 22, 51]. Evaluation_pre_ and evaluation_post_ were 10-minute written papers assessing 5 topics: “personal data” (T1), “prior knowledge” (T1), “user behaviour” (T1), “subjective evaluation of competencies” (T2), and “evaluation of e-learning” (T2). Answers were provided according to the type of question, either on a seven-point Likert scale (1 = strongly disagree, 7 = strongly agree), dichotomous questions (yes/no), or free responses. The response options were explicitly labeled to ensure clarity. No system logs data were collected with the aim of analysing user behaviour. The written theory papers of a total of 94 points across theory_pre_ + theory_post_ examined five areas of competence matching the defined learning objectives. These were “anatomy” (max. 11 points), “basic skills” (max. 29 points), “assignment tasks” (max. 6 points), and “normal findings / identifying structures in orientational section planes” (max. 48 points). In the assessment (theory_pre_ and thery_post_) there were proportionally overlapping and identical questions. Content validity was ensured by aligning questions directly with the e-learning material, learning objectives and validated educational frameworks (e.g., WINFOCUS reference planes) [21]. The point allocation for each section was determined during test development and aligned with the proportional representation of each topic in the e-learning module to ensure a fair weighting in the assessment. Questions required very short or multiple choice answers; exemplar questions are given in supplements 4 and 5. The theory-test was evaluated based on a solution key previously developed by experts. One point was awarded for each correct answer, while no negative points were given for incorrect responses. To confirm internal consistency, Cronbach’s alpha was calculated for all instruments. The assessments were administered under standardized conditions, without feedback between testing points, and without external guidance, thereby minimizing test-related bias.

### Statistical analysis

Data were collected using the survey and test tool LimeSurvey (LimeSurvey GmbH, Germany), written questionnaires, and practice exam sheets. All data were saved with Microsoft Excel. All statistical analyses were performed in Rstudio (Rstudio Team [2020]. Rstudio: Integrated Development for R. Rstudio, PBC, http://www.rstudio.com, last accessed on 20 04 2024) with R 4.0.3 (A Language and Environment for Statistical Computing, R Foundation for Statistical Computing, http://www.R-project.org; last accessed on 20 04 2024). Where possible, a main scale score was derived from the average of the subscale scores. Binary and categorical baseline variables are given as absolute numbers and percentages. Continuous data are given as mean and standard deviation (SD). Categorical variables were analyzed using the chi-squared test. Normality of data distributions was assessed using the Shapiro-Wilk test, ensuring appropriate use of parametric tests. Levene’s test was applied to check for homogeneity of variance. Paired t-tests were used to compare pre- and post-intervention scores within the study group, as the data were approximately normally distributed. This directly addresses the research question regarding subjective and objective competency gains through e-learning. For between-group comparisons (study group vs. reference group), post-test scores were compared using the Mann–Whitney U test, due to violations of normality assumptions in at least one group. This non-parametric test was chosen to examine differences in theoretical knowledge acquisition after e-learning between learners with different baseline experience levels. Additionally, independent samples t-tests were conducted where normal distribution and homogeneity of variance were confirmed. These analyses directly align with our research objective of assessing whether e-learning can effectively support learners across heterogeneous competency levels.

Parametric (ANOVA) and non-parametric (Kruskal-Wallis) analyses of variance were conducted, followed by pairwise post hoc tests (T-test or Mann-Whitney U test) to explore significant differences in responses across multiple participant subgroups. For multiple pairwise comparisons, the Bonferroni correction was used. P-values < 0.05 were considered statistically significant. Effect sizes were calculated using Cohen’s d. For validation purposes and to check the internal consistency of the scales used the Cronbach’s alpha values were calculated for the theoretical test (T1 and T2) and for the e-learning evaluation. For this study, a power analysis was performed to determine the sample size required to detect a statistically significant effect. Our power analysis, based on an expected effect size of 0.6 (derived from previous literature on e-learning in medical education [3, 26]), a significance level of 0.05, and a power of 0.80, was conducted using post hoc pairwise t-tests, resulting in a required sample size of 90 participants (45 per group). The actual sample size (*n* = 104; study group: 48, reference group: 56) exceeded this requirement.

## Results

### Group characterisation

A total of *n* = 104 data sets were included in the statistical analysis (*n* = 48 study group; *n* = 56 reference group). The demographic traits, previous clinical experience, user behaviour, and duration of preparation of the study and reference groups were analysed and are depicted in Table 1. The study group showed a lower mean age (*p* < 0.0001) and reported significantly less experience, specifically regarding “prior participation in ultrasound workshops” (*p* = 0.002), “echocardiographies seen so far” (*p* < 0.001), and “independently performed echocardiographies” (*p* < 0.0001). In the reference group, most participants were resident physicians (87.5%) and working in internal medicine (67.9%).

**Table 1:** Baseline characteristics, user behaviour and Preparation time in the study and the reference group

Items	Study Group (Students)	Reference Group (Physicians)	*p*-Value
Total number of registrations	*n* = 55	*n* = 65	
Approved participants for the workshop			
Not meeting the inclusion criteria (First state examination completed, use of educational materials, full participation in evaluations and tests)	*n* = 7	*n* = 9	
Participants included in the analysis	*n* = 48	*n* = 56	
**T1**			
	Mean ± SD	Mean ± SD	
**Age**	25.4 ± 3.3	31.7 ± 5.4	**< 0.0001**
**Gender**	N (%)	N (%)	1.0
*Female*	30 (62.5%)	36 (64.3%)	
*Male*	18 (37.5%)	20 (35.7%)	
*N/A*			
**Specialty (Physicians)**	N (%)	N (%)	
*Internal medicine*		38 (67.9%)	
*General medicine*		6 (10.7%)	
*Anaesthesiology*		8 (14.3%)	
*Other* *(Neurology*,* Paediatrics*,* Emergency medicine)*		4 (7.1%)	
**Position (Physicians)**	N (%)	N (%)	
*Students*	48 (100.0%)	0 (0,0%)	
*Resident*		49 (87.5%)	
*Consultant*		2 (3.6%)	
*Senior*		1 (1.8%)	
*Head of department*		0 (0,0%)	
*Physician Assistant*		2 (3.6%)	
*Other*		2 (3,6%)	
**Previous participation in ultrasound courses**	N (%)	N (%)	**0.002**
No	29 (60.4%)	17 (30.4%)	
Yes	17 (35.4%)	38 (67.9%)	
N/A	2 (4.2%)	1 (1.8%)	
**Shadowed/performed an ultrasound?**	N (%)	N (%)	
Abdomen	36 (54.5%)	48 (49.0%)	0.190
Head and neck	11 (16.7%)	21 (21.4%)	0.077
Puncture	19 (28.8%)	29 (29.6%)	0.149
**Observed an echocardiography**	N (%)	N (%)	<0.001
No	19 (39.6%)	8 (14.3%)	
Yes	21 (43.8%)	48 (85.7%)	
N/A	8 (16.7%)	0 (0.0%)	
**Performed an echocardiography**	N (%)	N (%)	**< 0.0001**
No	38 (79.2%)	25 (44.6%)	
Yes	2 (4.2%)	31 (55.4%)	
N/A	8 (16.7%)	0 (0.0%)	
**T2**			
**How much time did you spend e-learning?**	N (%)	N (%)	0.28
*1–2 h*	3 (6.3%)	11 (19.6%)	
*2.5–4 h*	28 (58.3%)	33 (58.9%)	
*4.5–6 h*	15 (31.3%)	7 (12.5%)	
*6.5–8 h*	2 (4.2%)	3 (5.4%)	
*8.5–10 h*	0 (0.0%)	2 (3.6%)	
**Have you watched the videos in the e-learning?**	N (%)	N (%)	0.84
*Yes*	46 (95.8%)	53 (94.6%)	
*No*	2 (4.2%)	3 (5.4%)	
**Have you had any practical training in echocardiography since receiving the educational media?**	N (%)	N (%)	0.67
*No*	27 (56.3%)	30 (53.6%)	
*Yes*	21 (43.8%)	26 (46.4%)	
**If applicable**,** to what extent have you had practical training?**	N (%)	N (%)	0.3
*1–4 h*	13 (61.9%)	16 (61.5%)	
*5–10 h*	8 (38.1%)	6 (23.1%)	
*11–20 h*	0 (0.0%)	1 (3.8%)	
*> 20 h*	0 (0.0%)	3 (11.5%)	

A majority in both groups had spent 2.5 to 4 h preparing for the workshop (study 58.3% vs. reference58.9%, *p* = 0.28). Most had also watched the videos in the e-learning (study 95.8% vs. reference 94.6%, *p* = 0.84). Multiple participants in both groups (study group 43.8%; referencegroup 46.4%) had, since receiving the educational media, “practical training in echocardiography” (*p* = 0.67).

### Subjective level of competency and evaluation of the E-Learning

**Fig. 2:**
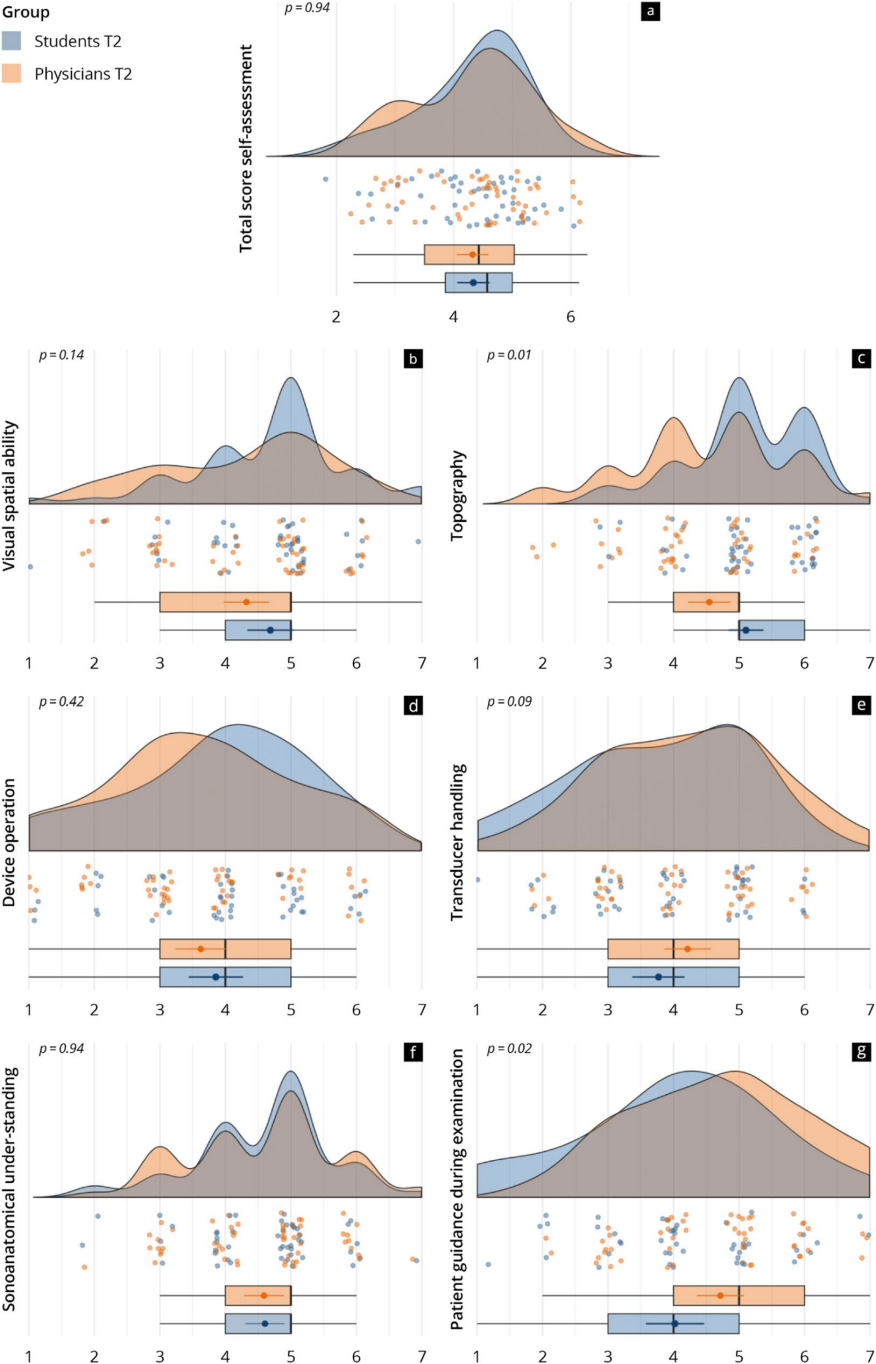
Resulting subjective levels of competencies in the study (blue) and reference group (orange) in the total score **(a)** as well as in the subcategories **(b-g)**. The raincloud plots visualise the data at T2

The subjective levels of competency in the study and the reference group at T2 are depicted in Fig. 2. Both groups estimated their competencies at similar levels after the preparation period (study 4.3 ± 0.9 vs. reference 4.3 ± 1.0; *p* = 0.94). The study group reported higher scores in the subcategory “topography” (*p* = 0.01), whereas the reference group indicated a higher subjective level of competence in “handling the patient during the examination”.

**Fig. 3:**
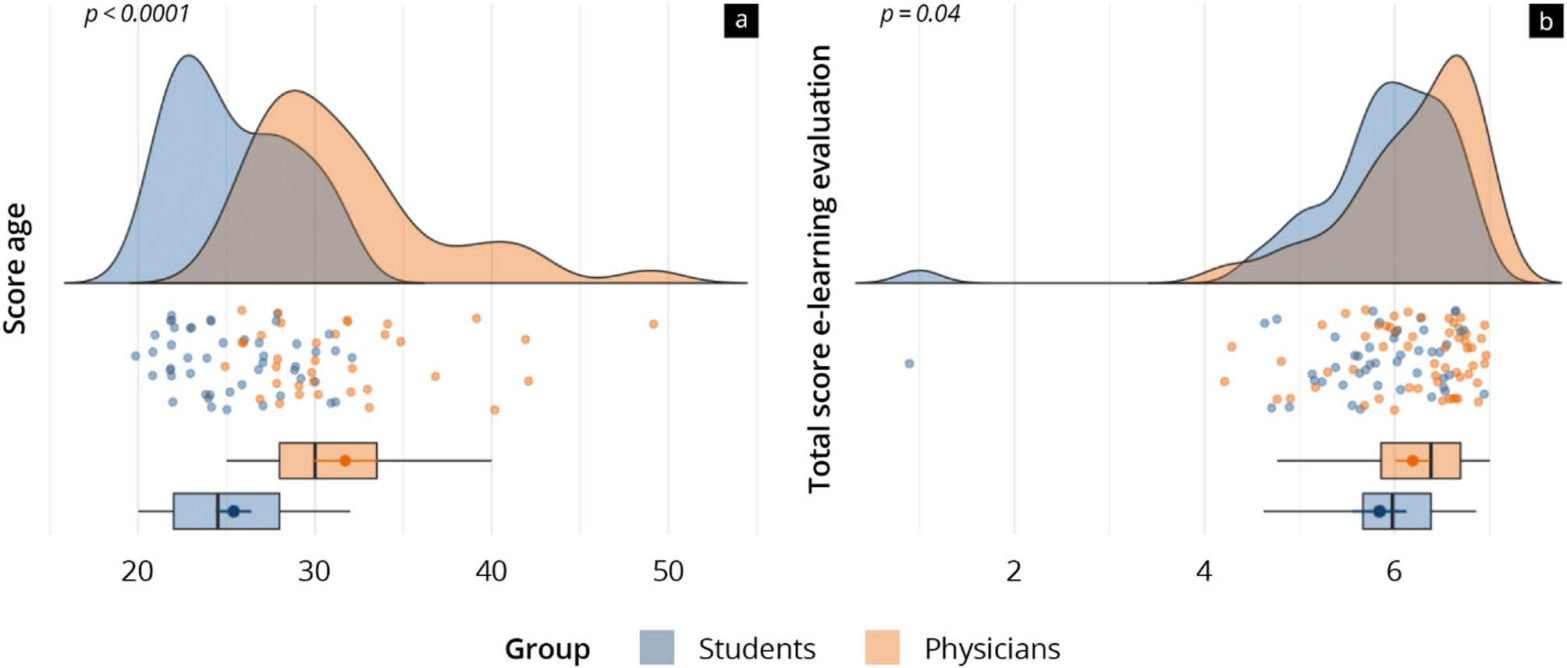
Age of the user groups **(a)** and total evaluation results of the e-learning **(b)** at T2. Data is presented as raincloud plots (blue = study group; orange = reference group)

The results of the evaluation of the e-learning can be found in Fig. 3; Table 2. Both groups rated the e-learning markedly positively (4.1–6.8 scale points). Altogether, the reference group gave a significantly higher rating than the study group (study 5.8 ± 0.9 vs. reference 6.2 ± 0.7; *p* = 0.04). This higher score was true for the subitems “structure of the menu” (*p* < 0.01), “study cards structure” (*p* = 0.03), “coverage of study cards” (*p* = 0.02), and “labelling of graphics” (*p* = 0.01), as well as “recommendation” (*p* < 0.001). Particularly highly rated (scale points 5.2–5.8) in both groups were “technique”, “handling of the menu”, “video explanations”, “learning progress”, “interactive features”, and “recommendation”. A little lower (scale points 4.0–5.0) were the ratings for “labelling of graphics”, “quantity of educational videos”, and “text-graphic relation”. The Cronbach’s alpha for the e-learning evaluation was 0.90, thereby indicating excellent internal consistency.

**Table 2:** Results of the evaluation of the e-learning by students and physicians

	Study Group (*n* = 48)	Reference Group (*n* = 56)	*p*-Value
	Mean ± SD	Mean ± SD	
**Total value**	5.8 ± 0.9	6.2 ± 0.7	0.04
Log-in function	4.7 ± 1.3	5.0 ± 1.3	0.34
Technique	6.8 ± 0.9	6.6 ± 1.0	0.42
Menu structure	5.5 ± 1.6	6.4 ± 0.9	0.002
Menu operability	6.2 ± 1.5	6.5 ± 1.1	0.15
Duration of videos	4.7 ± 1.3	5.1 ± 1.0	0.11
Quality of videos	5.1 ± 1.0	5.0 ± 1.2	0.55
Quantity of videos	4.1 ± 1.0	4.2 ± 0.9	0.95
Video explanations	5.4 ± 1.2	5.8 ± 1.5	0.14
Learning progress	5.2 ± 1.3	5.7 ± 1.1	0.07
Structure study cards	4.4 ± 1.4	5.0 ± 1.2	0.03
Workload per study card	4.4 ± 1.4	5.0 ± 1.0	0.02
Font size	4.6 ± 0.6	4.5 ± 0.8	0.32
Text volume	4.4 ± 0.9	4.5 ± 1.0	0.86
Graphic dimensions	4.5 ± 0.8	4.4 ± 1.0	0.53
Graphic labels	4.0 ± 1.3	4.6 ± 0.8	0.01
Quantity of graphics	5.3 ± 0.9	5.4 ± 1.0	0.66
Relation (text/graphic)	4.4 ± 0.7	4.5 ± 0.9	0.84
Design	5.1 ± 1.1	5.3 ± 1.1	0.38
Interactive features	6.0 ± 1.2	6.1 ± 1.3	0.97
Recommendation	5.3 ± 1.4	6.2 ± 0.8	< 0.001

### Objective development of competencies

**Fig. 4:**
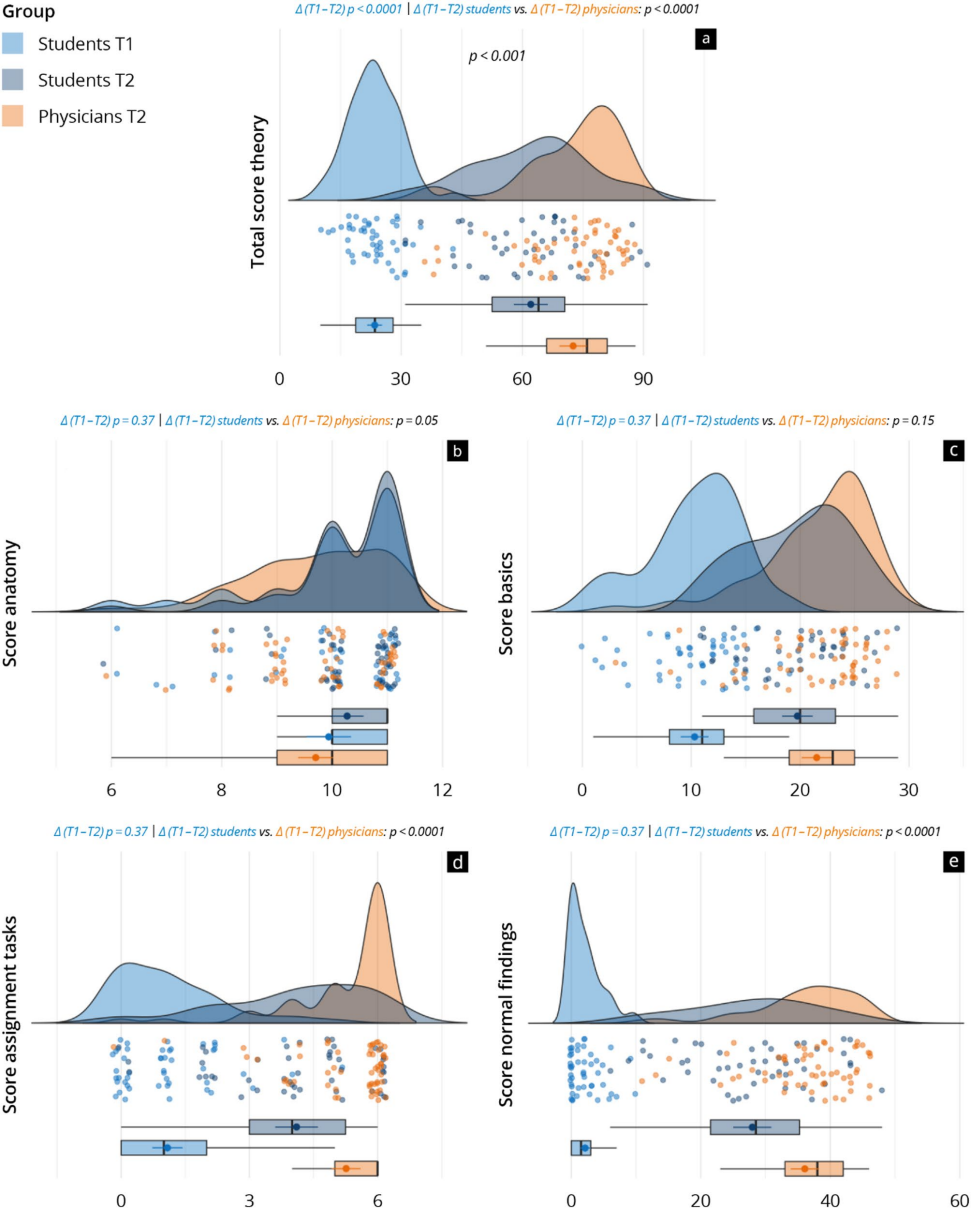
Results of the theory test of study and reference group at T1 and T2 in the total score **(a)** as well as in the subcategories **(b–e)** The raincloud plots visualise the results at T1 (light blue = study group) and T2 (dark blue = study group; orange = reference group)

The results of the theory tests in both the study and reference groups are depicted in Fig. 4 and supplement 6. From T1 to T2, the study group achieved a significant increase in total competencies (T1: 23.5 ± 6.3 vs. T2: 62.1 ± 14.6; *p* < 0.001) with a large effect (Cohen’s d = 3.43, 95% CI [2.68, 4.18]), and in the subitems “basics” (*p* < 0.001), “assignment tasks” (*p* < 0.001), and “normal findings” (*p* < 0.001). However, no improvement was noted in the subitem “anatomy” (*p* = 0.37). The reference group, which had more previous practical experience (*p* < 0.001), achieved in summary significantly higher scores at T2 (study 62.1 ± 14.6 vs. reference 72.6 ± 12.7; *p* < 0.001), also in the subitems “assignment tasks” (*p* < 0.001) and “normal findings” (*p* < 0.001). In “anatomy” (*p* = 0.05) and “basics” (*p* = 0.15), both groups showed similar levels of competency at T2. Cronbach’s alpha for the theory test was 0.77 at T1, indicative of acceptable internal consistency, and 0.88 at T2, indicative of good internal consistency.

### Factors of influence

The analysis and examination of possible influencing factors can be found in Table 3. Multivariate linear regression analysis showed that the factor “Performed an echocardiography – yes” (β = 14.9; *p* = 0.01) significantly influenced the theory test at T2.

**Table 3:** Parameters influencing the development of objective competencies in the study group (Students) and the control group (Physicians)

Items	Score Theory Test^post^ (study group)	Score Theory Test^post^ (control group)	*p*-value
**Previous participation in ultrasound courses**			
No	(*n* = 29) 60.1 ± 13.0	(*n* = 17) 71.4 ± 16.0	0.02
Yes	(*n* = 17) 65.6 ± 16.9	(*n* = 39) 73.1 ± 11.4	0.11
Intra-group comparison	0.26	0.69	
**Observed an echocardiography**			
No	(*n* = 19) 58.9 ± 11.7	(*n* = 8) 67.1 ± 19.4	0.29
Yes	(*n* = 21) 64.6 ± 16.7	(*n* = 48) 73.5 ± 11.4	0.03
Intra-group comparison	0.22	0.39	
**Performed an echocardiography**			
No	(*n* = 38) 61.1 ± 14.4	(*n* = 25) 68.9 ± 14.4	0.04
Yes	(*n* = 2) 77.5 ± 13.4	(*n* = 31) 75.6 ± 10.7	0.87
Intra-group comparison	0.321	0.06	
**How much time did you spend e-learning?**			
*1–2 h*	(*n* = 3) 40.7 ± 8.5	(*n* = 11) 72.09 ± 14.9	0.003
*2.5–4 h*	(*n* = 28) 62.8 ± 13.1	(*n* = 33) 73.6 ± 10.2	0.001
*4.5–6 h*	(*n* = 15) 64.3 ± 16.2	(*n* = 7) 66.7 ± 20.6	0.79
**Have you had any practical training in echocardiography since receiving the educational media?**			
Yes	(*n* = 19) 65.4 ± 12.4	(*n* = 26) 72.2 ± 12.2	0.08
*No*	(*n* = 29) 59.9 ± 15.6	(*n* = 30) 72.9 ± 13.4	0.001
Intra-group comparison	0.18	0.84	
**If applicable**,** to what extent have you had practical training?**			
*1–4 h*	(*n* = 13) 63.2 ± 12.8	(*n* = 16) 73.3 ± 9.5	0.02
*5–10 h*	(*n* = 8) 66.8 ± 13.1	(*n* = 6) 72.2 ± 18.2	0.55
Intra-group comparison	0.55	0.89	

An intergroup comparison with the aid of a differentiation test (binary scale) showed that the reference group tended to have significantly better results, particularly over those with the characteristics “Previous participation in ultrasound courses – no” (*p* = 0.02), “Observed an echocardiography - yes” (*p* = 0.03), “Performed an echocardiography – no” (*p* = 0.04) and “Have you had any practical training in echocardiography since receiving the educational media?” (*p* < 0.01). The time to prepare (“How much time did you spend e-learning?”) data collected suggested that the reference group could achieve significantly better results than the study group with a preparatory period of any length of up to 4 h (*p* < 0.01). This trend was not evident with a preparatory period of 4.5–6 h (*p* = 0.79). A better result in the test correlated positively with the duration of preparatory time (up to a preparatory period of 4 h) in both groups.

## Discussion

The primary objective of this study was to assess the extent to which a preparatory e-learning module could enhance the theoretical FOCUS competencies of a study group of medical students undertaking a training workshop. The study also aimed to compare the objective and subjective competency gain in this study group against those of a reference group (control) consisting of physicians, assessing competency gain across different clinical experience levels. Finally, participants evaluated the quality and acceptance of the e-learning.

The results suggest a significant improvement in the objective theoretical competencies of the study group following self-directed preparation. Although the reference group achieved significantly higher overall objective competency levels, both groups reported similarly high levels of subjective competency. Both groups similarly positively evaluated the e-learning platform, with the reference group providing a slightly higher overall rating.

Given the increasing cross-disciplinary application of FOCUS in emergency settings and for specific clinical assessments of cardiovascular status, the need for comprehensive training in this area is growing [40, 41, 56]. This study contributes valuable evidence supporting e-learning as a flexible and practical teaching method, aiming to foster innovative approaches in medical education.

### Discussion of objective competencies

Ongoing research has attempted to measure the effects of e-learning on theoretical competency growth in sonography [1–3, 5, 7, 8, 10, 17, 20, 27]. As suggested in previous studies [25, 30, 37], participants of our study group saw a significant increase in their theoretical skills after completing the FOCUS-specific e-learning. This was particularly observable in “basics”, “assignment tasks”, and “normal findings”. In “anatomy”, participants already had a high level of competency at T1, which explains the slight, statistically insignificant increase at T2. Conjecturally, study subjects might have benefitted from persisting anatomic and physiological knowledge from the preclinical stage. Studies have previously correlated ultrasound instruction and improvements in anatomy knowledge, though the courses in these studies were specifically designed and used to teach cardiac anatomy and physiology itself, unlike the workshop in our study [30, 37, 46]. Our study benefitted from using multiple-choice as well as very short answer questions, as these enabled clearer measures and more decisive assessment of competency growth in comparison with other studies [9, 47].

A defining characteristic of our study was the use of a reference group of physicians (control) who similarly used the FOCUS e-learning in preparation for a one-day FOCUS workshop. Prior studies of e-learning specialised on FOCUS tend to have one study group made of inexperienced participants including both students as well as physicians, with some using and some eschewing a control group [2, 25, 39, 46, 47]. Other studies used alternative teaching methods as a control, whether peer teaching, self-guided study, or team-based learning [9, 25]. Unlike other studies that have focused solely on students, we introduce a comparative perspective by including both students and physicians, helping to contextualise the effectiveness of e-learning across different learner groups. The results of this study and prior studies suggest that e-learning is not only a promising approach by which learners can acquire new theoretical competencies, but that e-learning is also effective for deepening and revising existing knowledge and skills, especially in ultrasound basics [20, 25, 30, 38]. This is particularly supported by the significant improvements observed in basic theoretical competences, especially in the subcategories “basics” and “normal findings”. After the preparation phase, the reference group showed a generally higher level of objective competencies, especially in “basics”, “assignment tasks”, and “normal findings”, though not in “anatomy”. This reiterates that the study group’s superior anatomical competencies may result from their recent pre-clinical training, where e-learning reinforces prior knowledge [25]. Several factors likely played a role in this difference, most notably the physicians’ greater clinical experience, as indicated by our regression analysis. Their more extensive hands-on exposure to echocardiography in daily practice likely gave them a stronger foundation for interpreting ultrasound images and performing related tasks [57–59]. E-learning was effective in improving theoretical competence and substantially narrowing the gap between novice and experienced learners, although, as expected, it was not able to close it entirely.

### Discussion of subjective competencies

Recent studies have examined both the objective and subjective increases in learner competencies achieved through e-learning [1, 8, 14, 17, 28, 30]. After the e-learning in this study, both groups estimated their level of skills similarly, suggesting a levelling of the subjective competency gradient. Prior research has also shown that participants with initially low self-assessments tend to reduce their lag during e-learning [14], a phenomenon attributed to the so-called “catch-up effect” [60].

The significantly higher self-assessment in the study group in the “topography” sub-category mirrors their objectively higher knowledge of anatomy. Meanwhile, the reference group estimated themselves higher in “patient handling during the examination”, which is explicable by their greater time spent in clinical practice and patient care. The discrepancies in subjective vs. objective competencies gained may reflect differences in the learning experiences of both groups: the study group may have overestimated their knowledge due to the theoretical focus, a phenomenon that can be explained by the Dunning–Kruger effect [61]. The reference group, benefiting from more clinical experience, gave a more realistic self-assessment. This suggests that subjective evaluations are influenced by individual experiences and learning strategies, which may not always align with objective competency development.

Overall, the results indicate that e-learning enhances educational success and positively influences learners’ subjective evaluation of their competencies. E-learning can, therefore, be a good tool to enhance target-oriented competency growth.

### Discussion of the evaluation of the E-learning

The evaluation of digital learning formats is an important part of quality management, and the value of qualitatively effective e-learning is also emphasised in the literature [1, 10, 20]. Following previous studies, we used a questionnaire to evaluate the learners’ perception of the quality of our e-learning [7, 14, 28, 30]. The evaluation of competencies was based on the Kirkpatrick model, focusing on competence development and participant feedback [62].

The e-learning was generally rated highly positively, especially in “technique”, “user-friendliness”, and “interactive features”, which together form the foundation of user-friendly e-learning software. To enhance the user experience further, we propose integrating individualized learning paths, which would tailor content to the diverse needs and preferences of learners. This approach, supported by feedback, could improve user-friendliness and foster a more personalized learning journey, thereby optimizing overall competency gain. The evaluation by the reference group, who had a significantly higher mean age, was significantly higher than the evaluation by the study group. This could be due to the study group’s higher expectations from digital teaching media as participants in the study group could be considered “digital natives”. These differences in expectations highlight an opportunity to develop even more individualised e-learning offers.

Optimisation is also important to ensure ongoing quality. For example, the waterfall model could advance the implementation and revision process of the e-learning by enabling systematic improvements through structured, sequential phases [55, 63]. In prior successful e-learning studies, special emphasis was placed on correcting all errors, adapting to the current scientific standard, optimising features, and implementing extensions [55, 63].

We propose to adapt the e-learning in our study after student evaluation to improve its quality. Feedback indicated an indirect wish for more videos: the quantity of videos was rated as “moderate”, while their quality and explanation were rated as “above average”. This suggests that participants value the clarity and depth of video content, but would benefit from an increased quantity to further enhance their learning experience. Prior studies confirm that video-based education is increasing in use and is popular among students, but they also show that instructional videos in isolation are insufficient and more interactive formats are desirable for competency gain and to meet learner preferences [9, 15, 25, 64]. These findings reinforce that no single method leads to successful learning but rather a combination of different teaching methods seems to be the most effective way to achieve broad growth in competencies [9, 10, 15, 20, 38].

In addition to measuring learning success, this study also investigated e-learning acceptance by users. E-learning acceptance was assessed using a multi-dimensional Likert-scale questionnaire that measured aspects such as technical usability, content quality, interactivity, and willingness to recommend the e-learning module. The positive feedback from the participants strongly suggests that the e-learning module was well received and accepted, implying not only positive reception but also overall acceptance of the e-learning platform. For this reason, the subjective data further promote e-learning concepts in future ultrasound teaching, and also to further develop it.

### Outlook of E-learning

While the creation of quality e-learning is resource-intensive, e-learning has clear benefits for sonography training [10, 20, 38]. E-learning is effective, flexible, motivating, and sustainable for learners and educators [1–3, 7, 8, 17, 20, 25, 27, 38]. Numerous studies including our own suggest that participants in e-learning develop competencies [3, 17, 25]. Digital instruction also enables flexibility in the time and place of learning, and to meet the varying preferences of learners themselves, so that personalised and demand-dependent learning can be assured [1, 7, 20, 38]. This flexibility can be especially useful for physicians with a high workload, but it is also beneficial for students [10, 20]. One study showed that even a very short e-learning course can be a useful preparation tool for ultrasound examinations [14, 27]. The scope and complexity of the course can vary depending on the topic, allowing e-learning to best suit the learning goal. In the future, e-learning could potentially facilitate individualised learning concepts that can be customised to the learner’s specific needs [65]. This could be done according to pre-tests and learning preferences, or to better tailor courses to specific learning needs or disabilities [65]. The use of AI could enhance this development [66].

E-learning formats such as the one developed in this study can be expanded step-by-step, such as by including pathologies with independently created content, or by incorporating links to pathology atlases. Digital toolkits for the construction of websites can simplify structuring web pages and the realisation of e-learning formats, while high-quality e-learning courses built with such tools have been shown to motivate participants, generate learner interest, and gain wide acceptance [1, 8, 14, 17]. In the future, system logs could be used for a more detailed evaluation of user behaviour, allowing for a more precise monitoring of the hours spent on the platform and the modules worked on, thus allowing for a more differentiated and objective analysis of e-learning.

As shown by our data and in previous studies, e-learning can stably and reliably build up basic knowledge and skills [2, 25, 30, 39]. The sustainable digitalisation of in-person formats could lead to a decreased need for personnel and spatial resources [38] and could lead to a more effective use of in-person course time. The results suggest that e-learning effectively facilitates the acquisition of foundational theoretical knowledge, but additional practical components may be needed to fully address the needs of novice learners. Previous studies have also suggested that e-learning can contribute to the development of practical skills [14], though it cannot entirely replace in-person practical training [2, 9].

In summary, our data reflects favourably on integrating e-learning into blended learning teaching of FOCUS, as is also suggested by prior studies [20, 28, 30, 67]. To support under-resourced regions and promote efficient resource use, specialist societies should consider developing recommendations and clear guidelines for these courses. Furthermore, these societies should consider accrediting e-learning to enable consistent quality of training and to develop the format to its full potential for learners [10, 14].

### Limitations

This study has several limitations that should be considered when interpreting the findings.

First, there were some systematic differences between study and reference group. The lack of an in-person introductory session for the reference group (including the pre-test) limits the ability to determine whether their higher post-test scores reflect learning from the e-learning module or pre-existing knowledge. To ensure validity, the written test was not conducted at home to prevent external aids. The reference group had greater age, clinical experience, prior examinations, and FOCUS exposure, likely contributing to their stronger performance. This finding is supported by a trend in the intergroup comparison, which showed that within the reference group, participants who had already performed echocardiography scored better than those who had not (*p* = 0.06). While these influencing variables were considered in the interpretation of results and included in a regression analysis, the subgroup sizes were small, and the comparisons should be interpreted as indicative trends rather than definitive effects. Preparation time differed between groups: the study group had a fixed 1–2 week period, whereas the reference group had up to four months to engage with the material. Although this variation might have allowed deeper engagement for some physicians. The reference group was slightly larger than the study group, with a difference of eight participants. Although this discrepancy is relatively small, it may still have influenced group comparisons. Overall, this limits the interpretation of comparative effects, which means that, as stated earlier, physicians should primarily be interpreted as a reference group.

Second, the assessments (theory_pre_ and theory_post_) contained overlapping and partially identical questions, introducing the possibility of test-retest bias. This was mitigated by a time delay between tests and by withholding feedback after the pre-test, but some recall effects cannot be ruled out.

Third, this study focused exclusively on theoretical knowledge acquisition, assessed through objective tests and subjective evaluations. The assessment of practical competencies was not feasible due to logistical and methodological constraints (e.g., lack of standardized evaluation conditions across multiple sites, need for expert raters and time-intensive evaluation processes). Consequently, the results do not allow conclusions about the acquisition of practical competencies. Future studies should include formats such as Direct Observation of Procedural Skills (DOPS) or Objective Structured Clinical Examinations (OSCEs) to evaluate the effect of e-learning on practical competencies.

Fourth, only students who had completed the first state medical examination were eligible, and thus the findings cannot be extrapolated to earlier-semester students or other groups such as nurses or allied health professionals, which may limit generalizability to other educational settings or learner populations.

Fifth, although test administration was standardized, self-selection bias remains possible. Participants who chose to take part may have been more motivated or more comfortable with digital learning formats, potentially leading to an overestimation of the e-learning module’s effectiveness.

Finally, the study did not include a second control group without e-learning access (e.g., textbook-only preparation), which would have enabled a more nuanced evaluation of the added value of e-learning compared to analog learning strategies. Furthermore, while participants had access to the e-learning platform after the initial course, no follow-up data were collected to assess long-term competency retention.

## Conclusion

The present study highlights the potential of e-learning in enhancing theoretical ultrasound competencies among those undertaking FOCUS training. However, while e-learning proves effective in improving theoretical knowledge, it cannot fully compensate for the clinical experience gained through hands-on practice. The users’ positive evaluation of e-learning underscores its suitability and acceptance as a valuable tool in ultrasound education. Digital learning media, such as e-learning platforms, should therefore be more prominently integrated into future ultrasound training programs. We propose that clear guidelines for the certification of e-learning platforms, focusing on content quality, user engagement, and assessment standards, be developed in alignment with existing ultrasound education frameworks. However, further investigations may be needed to examine these relationships in more depth and clarify their underlying mechanisms. Additionally, certification and accreditation by professional societies could ensure a modern, innovative, and forward-looking ultrasound educational practice that aligns with evolving technological advancements and educational needs.

## Electronic supplementary material

Below is the link to the electronic supplementary material.

Supplementary Material 1

Supplementary Material 2

Supplementary Material 3

Supplementary Material 4

Supplementary Material 5

Supplementary Material 6

## Data Availability

Data cannot be shared publicly because of institutional and national data policy restrictions imposed by the Ethics committee since the data contain potentially identifying study participants’ information. Data are available upon request from the Johannes Gutenberg University Mainz Medical Center (contact via weimer@uni-mainz.de) for researchers who meet the criteria for access to confidential data (please provide the manuscript title with your enquiry).
