# Supplementary material for: The effectiveness of e-learning in focused cardiac ultrasound training: a prospective controlled study
Source: BMC Med Educ. 2025 May 30;25:806. doi: 10.1186/s12909-025-07409-y (PMC12125877; doi:10.1186/s12909-025-07409-y)
Supplement: Supplementary file 1 — Supplementary Material 1 [file 12909_2025_7409_MOESM1_ESM.pdf]

Sample question 1

Describe the **anatomical picture** as accurately as possible.

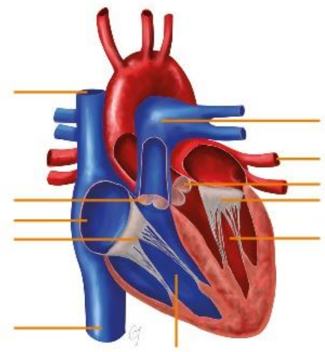

Sample question 2

Label the **ultrasound windows / axes** shown in the diagram with the correct terms.

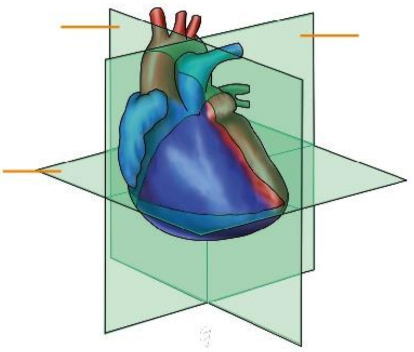

Sample question 3

Which **artefacts** are indicated by the arrows?

1. Artefact:
2. Artefact:

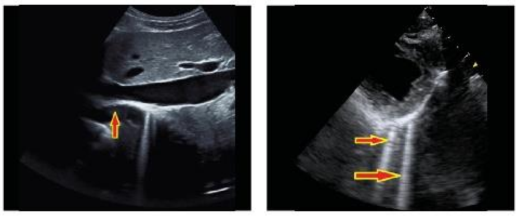

Sample question 4

Which **image modes** are shown in the pictures?

1. Image mode:
2. Image mode:

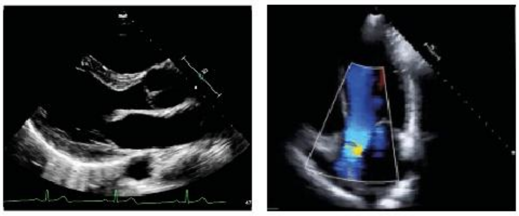

Sample question 5

· Complete the following sentence: The higher the frequency, the ______ the depth of penetration.

· Complete the following sentence: The lower the frequency, the ______ the depth of penetration.

Sample question 6

· Complete the following sentence: The correct sonomorphological term for "dark" areas in an image is: ______

· Complete the following sentence: The correct sonomorphological term for "light" areas in an image is: ______

Sample question 7

What **transducers** are shown here?

1: 2: 3:

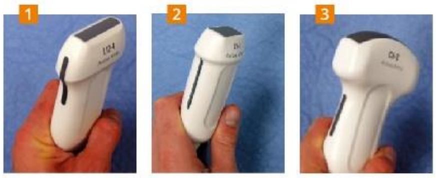

Supplement 4 Sample Questions of the Theory Test (The "Basic Skills" Area of Competency)
Very short answer questions were used to evaluate competencies in the areas of anatomical knowledge (sample questions 1 + 2); the understanding of artefacts and image modes (sample questions 3 + 4); as well as physical and accordingly ultrasound basics, including the different probes (sample questions 5 – 7).
