# Supplementary material for: The effectiveness of e-learning in focused cardiac ultrasound training: a prospective controlled study
Source: BMC Med Educ. 2025 May 30;25:806. doi: 10.1186/s12909-025-07409-y (PMC12125877; doi:10.1186/s12909-025-07409-y)
Supplement: Supplementary file 2 — Supplementary Material 2 [file 12909_2025_7409_MOESM2_ESM.pdf]

Sample question 8

Match the **transducer image** shown here with the **corresponding ultrasound image**.

1: ☐ 2: ☐ 3: ☐ 4: ☐ 5: ☐

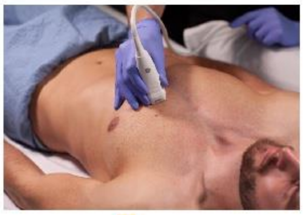

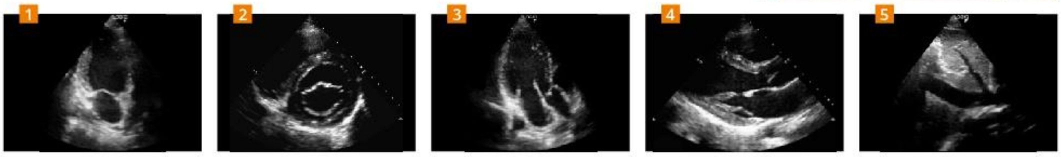

Sample question 9

Match the **transducer image** shown here with the **corresponding ultrasound image**.

1: ☐ 2: ☐ 3: ☐ 4: ☐ 5: ☐

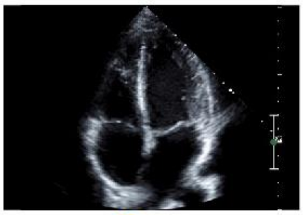

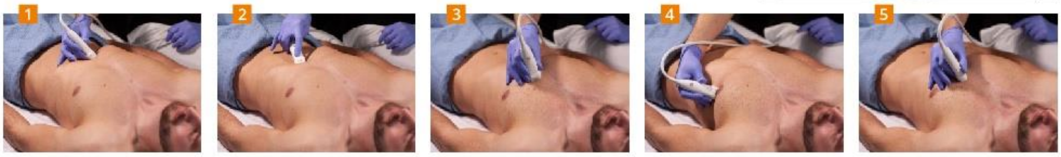

Sample question 10

Normal findings: What structures are marked in the image? Name the **sectional planes** and describe them as accurately as possible!

1:	1:
2:	2:
3:	3:
4:	4:
5:	5:

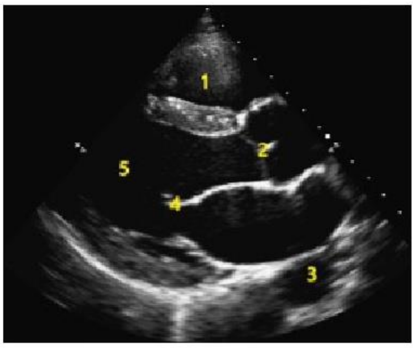

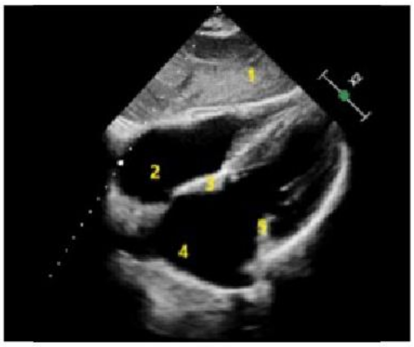

Supplement 5 Sample Questions of the Theory Test (The "Assignment Tasks" and "Normal Findings/ Identifying Structures in Orientational Section Planes" Areas of Competency)

The single choice questions used (sample questions 8 + 9) tested the understanding of probe positioning and the corresponding image. With the aid of very short answer questions (sample question 10), we examined the theoretical sonoanatomical knowledge by having test takers name structures in echocardiographic orientational sections in the B-mode.
