# Supplementary material for: The effectiveness of e-learning in focused cardiac ultrasound training: a prospective controlled study
Source: BMC Med Educ. 2025 May 30;25:806. doi: 10.1186/s12909-025-07409-y (PMC12125877; doi:10.1186/s12909-025-07409-y)
Supplement: Supplementary file 3 — Supplementary Material 3 [file 12909_2025_7409_MOESM3_ESM.pdf]

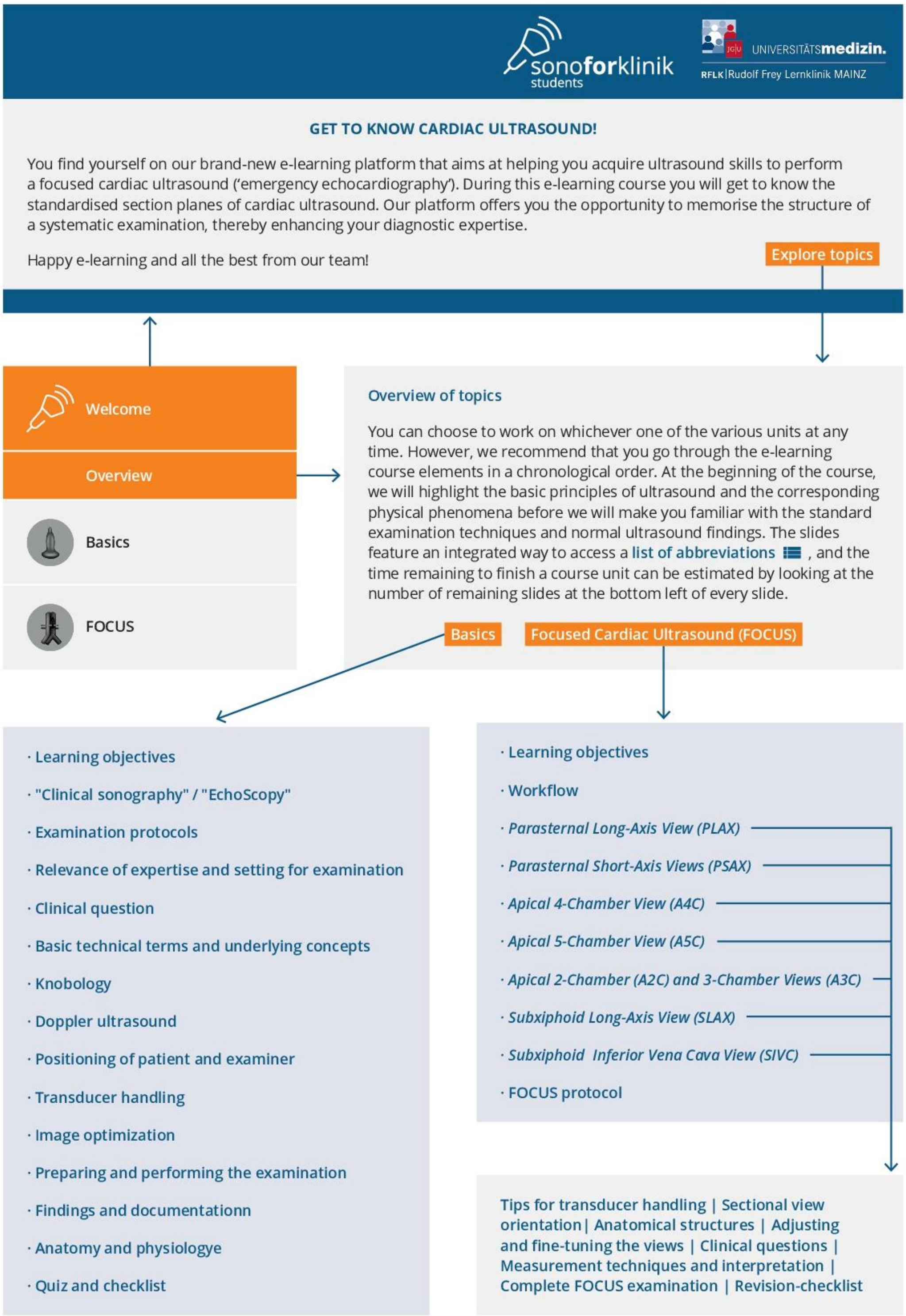

Supplement 1: The Structure of E-Learning Content

The e-learning units are accessed by clicking on them in the drop-down menu. Some units have a further drop-down menu for subchapters, which ensures a clear arrangement and manageability of the e-learning. Users can follow the pre-set order of the items, though this is not obligatory. Arrows in the graphics lead to the respective learning goals of the unit or subchapter. Relationships between subchapters and common features in the e-learning structure are always shown by arrows.
