# Supplementary material for: The effectiveness of e-learning in focused cardiac ultrasound training: a prospective controlled study
Source: BMC Med Educ. 2025 May 30;25:806. doi: 10.1186/s12909-025-07409-y (PMC12125877; doi:10.1186/s12909-025-07409-y)
Supplement: Supplementary file 4 — Supplementary Material 4 [file 12909_2025_7409_MOESM4_ESM.pdf]

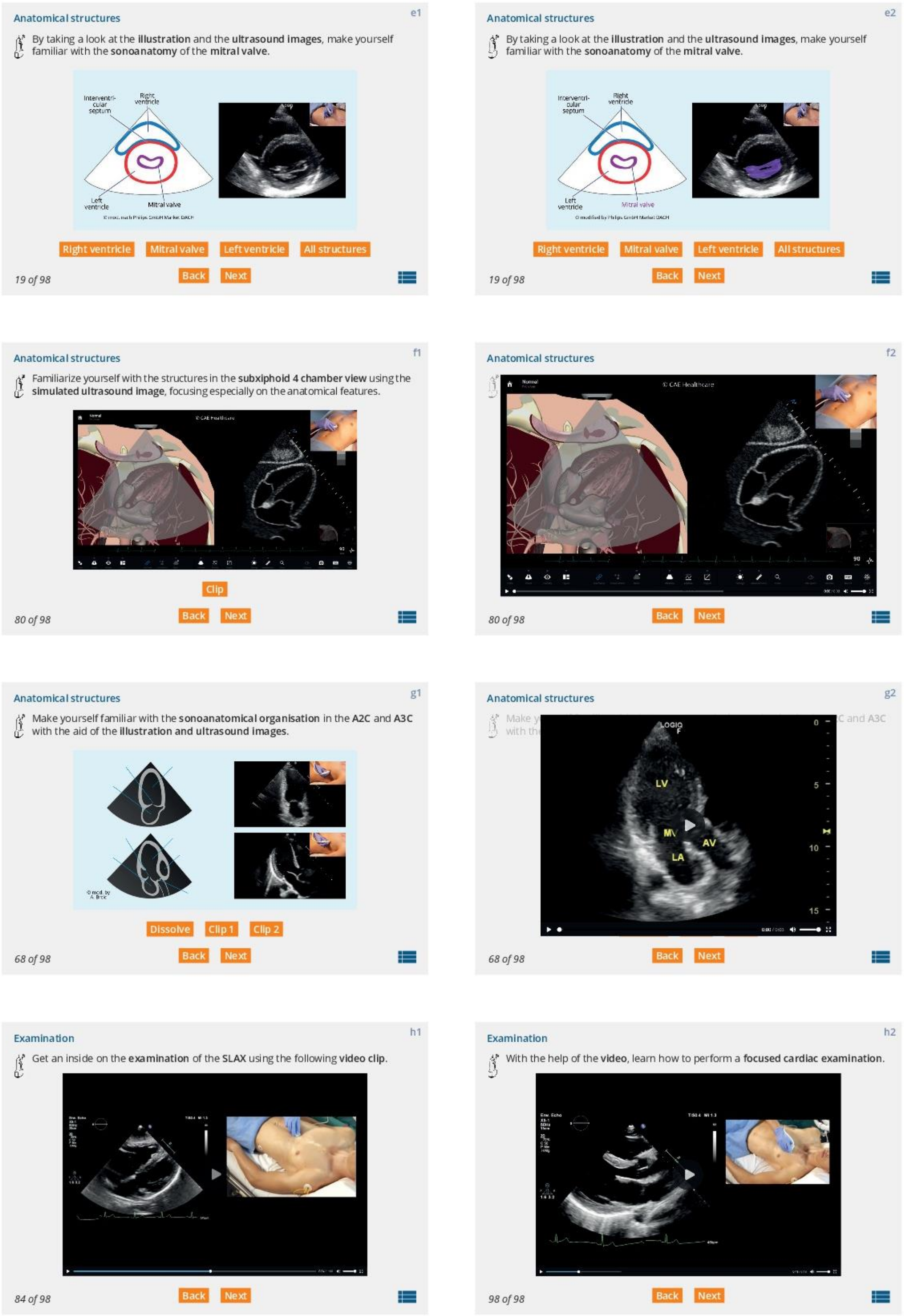

Supplement 3 Examples for Slides and Functions of the E-Learning, Part 2

First row (e1 + e2): This illustrates the most important click functions using the example of the PSAX mitral valve to better understand the sonoanatomical structure. There is a colour overlay for every important anatomical structure (e2).

Second row (f1 + f2): Further simulation pictures of the standard planes can help in building a better comprehensive knowledge (f1), thus click functions open clips of the ultrasound (f2). In this way, users can directly compare simulation images and real sonography on a patient.
Third row (g1 + g2): Here is an overview slide on how to get the apical two- and three-chamber views, including a schematic with the ultrasound image and respective positioning of the probe (g1). By clicking on them, the user can view clip sequences with anatomic labels (g2). A further click starts playing the clip.
Fourth row (h1 + h2): At the end of every unit, the user can watch videos on how to perform the examination. These videos include both depictions of handling the probe and the corresponding images received. The video explains the standard section plane with the relevant anatomy.
