# Supplementary material for: The effectiveness of e-learning in focused cardiac ultrasound training: a prospective controlled study
Source: BMC Med Educ. 2025 May 30;25:806. doi: 10.1186/s12909-025-07409-y (PMC12125877; doi:10.1186/s12909-025-07409-y)
Supplement: Supplementary file 5 — Supplementary Material 5 [file 12909_2025_7409_MOESM5_ESM.pdf]

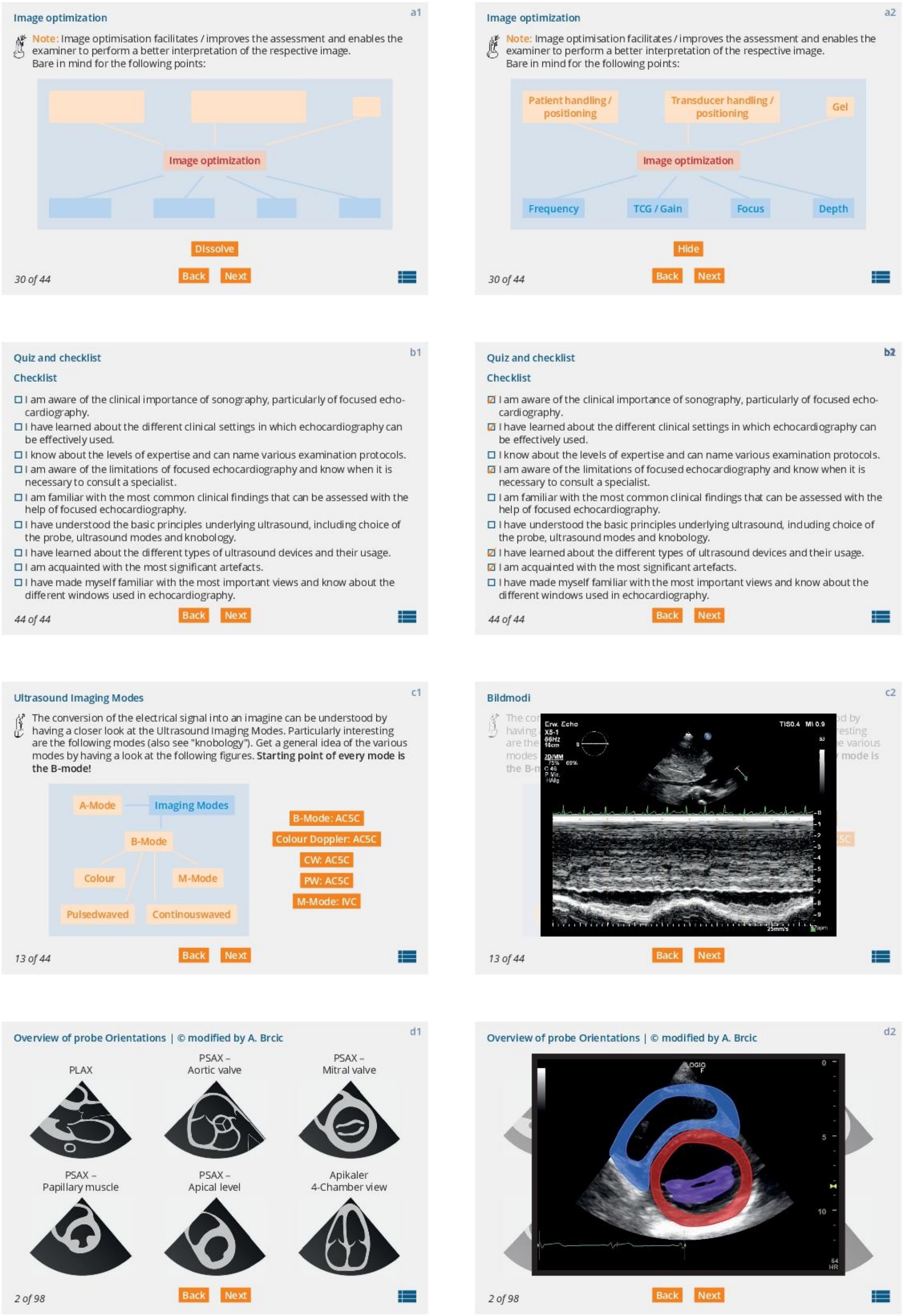

Supplement 2: Examples of Slides and Functions of the E-Learning, Part 1

First row (a1 + a2): Shown is how the solution appears when the “show answer” button is clicked (a “click function”).

Second row (b1 + b2): An exemplary checklist that serves as revision of the learned content and that can be used for self-assessment (“checking”).

Third row (c1 + c2): This slide serves as an overview of the different sonographic modes (c1), which lead to the sonographic illustrations when the relevant buttons are clicked (c2). Additionally, special

functions and measuring tools appear upon clicking the button at the right side of the slide (e.g. doppler mode).
Fourth row (d1 + d2): An overview of the most important standard section planes illustrated in schematics (d1). An overlay with anatomical structures can be accessed through further click functions.
