# Supplementary material for: The effectiveness of e-learning in focused cardiac ultrasound training: a prospective controlled study
Source: BMC Med Educ. 2025 May 30;25:806. doi: 10.1186/s12909-025-07409-y (PMC12125877; doi:10.1186/s12909-025-07409-y)
Supplement: Supplementary file 6 — Supplementary Material 6 [file 12909_2025_7409_MOESM6_ESM.pdf]

Supplement 6 Objective Development of Competencies in the Study Group (Students) and Level of Competencies in the Control Group (Physicians)

**Area of Competency**	**Score Theory Test^{pre} (study group)**	**Score Theory Test^{post} (study group)**	**p-Value study group**	**Score Theory Test^{post} (control group)**	**p-Value groups**
	MW ± SD	MW ± SD		MW ± SD	
**Total result**<br>**Max. 94 P**	23.5 ± 6.3	62.1 ± 14.6	**<0.0001**	72.6 ± 12.7	**<0.0001**
**Anatomy**<br>Max. 11P	9.9 ± 1.4	10.3 ± 1.0	0.37	9.7 ± 1.2	0.05
**Basic skills**<br>Max. 29P	10.3 ± 4.4	19.8 ± 4.8	**<0.0001**	21.5 ± 5.2	0.15
**Assignment tasks**<br>Max. 6P	1.1 ± 1.2	4.1 ± 1.7	**<0.0001**	5.3 ± 1.3	**<0.001**
**Normal findings/labelling sections**<br>Max. 48P	2.1 ± 2.5	28.0 ± 10.1	**<0.0001**	36.1 ± 8.2	**<0.0001**
